# Association of Beta-2 Microglobulin with Stroke and All-Cause Mortality in Adults Aged ≥40 in U.S.: NHANES III

**DOI:** 10.31083/j.rcm2402043

**Published:** 2023-02-02

**Authors:** Yanan Zhang, Xiaobing Zhai, Keyang Liu, Wenzhi Ma, Shiyang Li, Jing Zeng, Mei Yang, Feng Zhou, Bing Xiang, Jinhong Cao, Ehab S. Eshak

**Affiliations:** ^1^Research Center for Health Promotion in Women, Youth and Children, Hubei Province Key Laboratory of Occupational Hazard Identification and Control, School of Public Health, Wuhan University of Science and Technology, 430065 Wuhan, Hubei, China; ^2^Public Health, Department of Social Medicine, Osaka University Graduate School of Medicine, 565-0871 Osaka, Japan; ^3^Department of Epidemiology and Biostatistics, School of Health Sciences, Wuhan University, 430071 Wuhan, Hubei, China; ^4^Public Health and Community Medicine, Faculty of Medicine, Minia University, Mainroad Shalabyland, 61519 Minia, Egypt; ^5^Advanced Clinical Epidemiology, Medical Data Science Unit, Public Health Osaka University Graduate School of Medicine, 565-0871 Osaka, Japan

**Keywords:** beta-2-microglobulin, stroke, all-cause mortality, cohort study

## Abstract

**Background:**

Stroke is the predominant cause of death worldwide. We aimed to investigate the association of serum beta-2 microglobulin (β2M) concentrations with risk of stroke and all-cause mortalities in a cohort study.

**Methods:**

Overall, 4914 U.S. adults (mean age = 63.0 years, 44.3% male) were recruited from the National Health and Nutrition Examination Survey (NHANES Ⅲ). During a median follow-up of 19.4 years, 254 stroke deaths and 3415 all-cause deaths were identified by the National Center for Health Statistics. The associations of β2M with stroke and all-cause mortalities were investigated by using weighted Cox proportional hazard regression models.

**Results:**

β2M was positively associated with stroke and all-cause mortality in unadjusted models and multivariable-adjusted models. The multivariable HR (95% CI) for stroke mortality in Q5 VS Q1 of serum β2M concentrations was 3.45 (1.33–8.91; *p* for trend = 0.001) and that for all-cause mortality was 3.95 (3.05–5.12; *p* for trend < 0.001). In subgroup analyses, the association of β2M and stroke mortality did not vary by different levels of sociodemographic and general stroke risk factors (*p *_interaction_ > 0.05). In addition, the magnitude of positive association between β2M with all-cause mortality did vary by age, ratio of family income to poverty, smoking status, and history of hypertensive (*p *_interaction_ < 0.05).

**Conclusions:**

Our findings suggest that support that β2M may be a marker of stroke and all-cause mortality, which provides a new perspective for the study of cerebrovascular health and long-term survival in the future.

## 1. Introduction

Stroke, a leading cause of morbidity and mortality worldwide, is the third most common cause of death in most Western countries, after coronary heart disease and cancer [1]. New research shows that the incidence of stroke is increasing. One in four people in the world has experienced a stroke in their lifetime. It is also a major cause of disability worldwide, with 50 per cent of survivors suffering from chronic disability [2]. In a county-level study, stroke mortality among adults aged 35 to 64 in the United States increased from 14.7/100,000 in 2010 to 15.4/100,000 in 2016 [3]. At present, many researchers have explored the death factors of stroke, the retrospective study of Wa á kowicz shows that hypertension, smoking, coronary heart disease and previous stroke history are risk factors leading to death of patients with acute stroke [4]. Given the high incidence, disability, and mortality rates, and the high financial burden of stroke [5, 6, 7], there is great interest in finding novel markers that identify individuals at higher risk of stroke. One potential risk marker may be beta-2 microglobulin (β2M). β2M, a non-glycosylated protein that exists in each nucleus, which forms major histocompatibility complex (MHC) class I molecules on the cell surface [8].

Serum β2M levels elevate with systemic inflammation and deteriorated glomerular filtration rate (GFR), prompting β2M as a marker of renal disease and kidney function [9]. β2M is closely associated to the incidence and death of a variety of diseases, including cardiovascular (CVD) [10], Chronic Obstructive Pulmonary Disease (COPD) [11], chronic kidney disease (CKD) [9], diabetes [12], cancer [13] and hemodialysis mortality [14]. To further explore the role of β2M in overall health, several previous epidemiological studies showed the associations between β2M and all-cause and cause-specific mortality, and found higher β2M was significantly associated with higher risk of sudden cardiac death (SCD), end-stage renal disease (ESRD), and infectious mortality [15, 16, 17]. In addition, blood disease is one of the uncommon causes of cerebrovascular diseases such as acute stroke. The detection of hematological markers will help correct diagnosis of stroke. The positive correlation between β2M and acute stroke in this unusual cause is still lacking [18]. Some studies showed the association between β2M with CVD mortality [16, 19, 20] and stroke incidence [21, 22], however, no study further reported the association between β2M and specific stroke mortality. The evidence to determine the relationship between β2M and stroke mortality is still limited, and no prior study has specifically evaluated the prognostic value of β2M in adults aged ≥40 in U.S.

In this study, we aimed to examine the associations of β2M with stroke and all-cause mortality, using over 20 years of follow-up in the National Health and Nutrition Examination Survey III (NHANES III). Subgroup analyses were carried out, considering potentially traditional risk factors associated with stroke, including age, sex, ratio of family income to poverty, BMI, alcohol, smoking, history of hypertension, and history of diabetes [23, 24].

## 2. Methods

### 2.1 NHANES III

The Third National Health and Nutrition Examination Survey (NHANES III) is a large-scale, multistage, ongoing, nationwide probability sampling survey by National Center for Health Statistics (NCHS) during the period 1988–1994. NHANES III is a two-stage, six-year comprehensive survey, overall survey response rate of participants (78%) has been described in previous studies [25, 26]. All baseline data are available at https://wwwn.cdc.gov/nchs/nhanes/nhanes3/default.aspx.

### 2.2 Study Population

In NHANES III, blood samples from 7807 participants were measured for β2M content. In this study, we performed a prospective cohort of β2M levels with risk of stroke and all-cause mortality in the U.S. adult population. Participants of this study had not a history of stroke at baseline and had mortality follow-up information including underlying cause of death. Considering the onset age of stroke, we excluded those aged <40 years (n = 1083). We further excluded participants who had missing information on β2M (n = 670) and baseline characteristics (n = 814), or if self-reported heart failure or stroke onset (n = 456). The final analytic cohort consisted of 4914 participants. All participants provided written informed consent. The NHANES was approved by the NCHS Ethics Review Board.

### 2.3 Exposure Measurement and Outcome Assessment

In 2009, beta-2 microglobulin (β2M) levels were measured from stored surplus serum samples of NHANES III participants in the University of Minnesota, stored at –70 °C until the time of β2M measurement and was assayed using the N Latex β2 microglobulin assay, Siemens Diagnostics, and IL. The inter-assay coefficient of variation for the β2M assay was 2.7% (mean 1.757 mg/L) when β2M concentrations ranged from 0.253 to 61.700 mg/L. Given the possible nonlinear relationship of β2M levels with mortality rates, participants were divided into the following five categories based on quintiles of serum β2M levels: <1.73 (reference group), 1.73 to 2.00, 2.01 to 2.33, 2.34 to 2.90, and ≥2.91 mg/L. Stroke mortality includes deaths caused by ischemic stroke and hemorrhagic stroke, but there is no clear distinction between stroke in the data of NHANES III. Participants were asked if and when they had a stroke. For example, participants were asked: “Have doctors or other health professionals ever told you about a stroke?”. All causes mortality included stroke (codes I60–I69), heart disease (codes I00–I09, I11, I13, and I20–I51), cancer (codes C00–C97), and other causes (codes C98–C99). The causes of death were classified according to the codes of ICD-10 (International Statistical Classification of Diseases, 10th Edition) [27, 28]. NHANES participants were linked to the National Death Index through a probabilistic matching algorithm to determine mortality status and special causes of death as of December 31, 2015.

### 2.4 Covariates

Information on sociodemographic characteristics and potential biochemical factors was collected at baseline (n = 4914), including age (we categorized into <60 and ≥60 years), sex (male and female), race/ethnicity (non-Hispanic white, non-Hispanic black, Mexican American, and Other), marital status (married, widowed, divorced, and single), ratio of family income to poverty (we categorized into <1.58, 1.58–4.09, and >4.09), alcohol (we categorized into never drinker, moderate drinker, and heavy drinker), smoking (we categorized into never smoker, former smoker, and current smoker). At baseline, body mass index (BMI) was calculated by the formula: BMI = weight/height × height. Glycated hemoglobin (%), serum creatinine (mg/dL), low-density lipoprotein (LDL)-cholesterol (mg/dL), high-density lipoprotein (HDL)-cholesterol (mg/dL), c-reactive protein (mg/dL) were performed by the Laboratory at the University of Missouri and the University of Minnesota. GFR was estimated using the Chronic Kidney Disease Epidemiology Collaboration (CKD-EPI) 2009 creatinine equation [29]. Histories of hypertension and diabetes were recorded by participants’ self-reports.

### 2.5 Statistical Analysis

Trends of population characteristics across quintiles of β2M were examined by using Anova for continuous variables, or Chi-square tests for categorical variables. The hazard ratios and 95% confidence intervals between β2M with stroke and all-cause mortality were investigated by using weighted Cox proportional hazards regression, model 1: adjusted for age, sex, race/ethnicity, and marital status; model 2: model 1 + ratio of family income to poverty, BMI, alcohol, and smoking; model 3: model 2 + glycated hemoglobin (%), serum creatinine (mg/dL), LDL-cholesterol (mg/dL), HDL-cholesterol (mg/dL), c-reactive protein(mg/dL), GFR, history of hypertension and history of diabetes.

In addition, subgroup analyses were completed to assess the heterogeneity in the associations of β2M with risk of stroke and all-cause mortality and tested for interaction *p* value for age groups (<60 and ≥60 years), sex (male and female), ratio of family income to poverty (<1.58, and ≥1.58), BMI (<25, 25–29.9, and ≥30 kg/$m^{2}$), alcohol (current drinker and non-current drinker), smoking (current smoker and non-current smoker), history of hypertension (no and yes), and history of diabetes (no and yes).

To further test the robustness and potential variations of associations of β2M with stroke and all-cause mortality, we conducted several sensitivity analyses. First, we repeated all analyses by excluding deaths during the first two years of follow-up to reduce potential reverse causation. Second, we excluded individuals with chronic kidney disease, diabetes, or hypertension, because both β2M and stroke events could be influenced by major chronic diseases. Finally, we estimated the association between β2M levels and risk of stroke among those with GFR ≥60 mL⋅$\min^{-1}$ ⋅1.73 $m^{-2}$, the cutoff for abnormal renal function/chronic kidney disease. All analyses were conducted using the SAS software version 9.4 (SAS Institute, Cary, NC, USA) and GraphPad Prism 8 software (GraphPad Software, San Diego, CA, USA). Two-sided *p* values < 0.05 were considered statistically significant.

## 3. Results

### 3.1 Population Characteristics

**Fig. 1.**
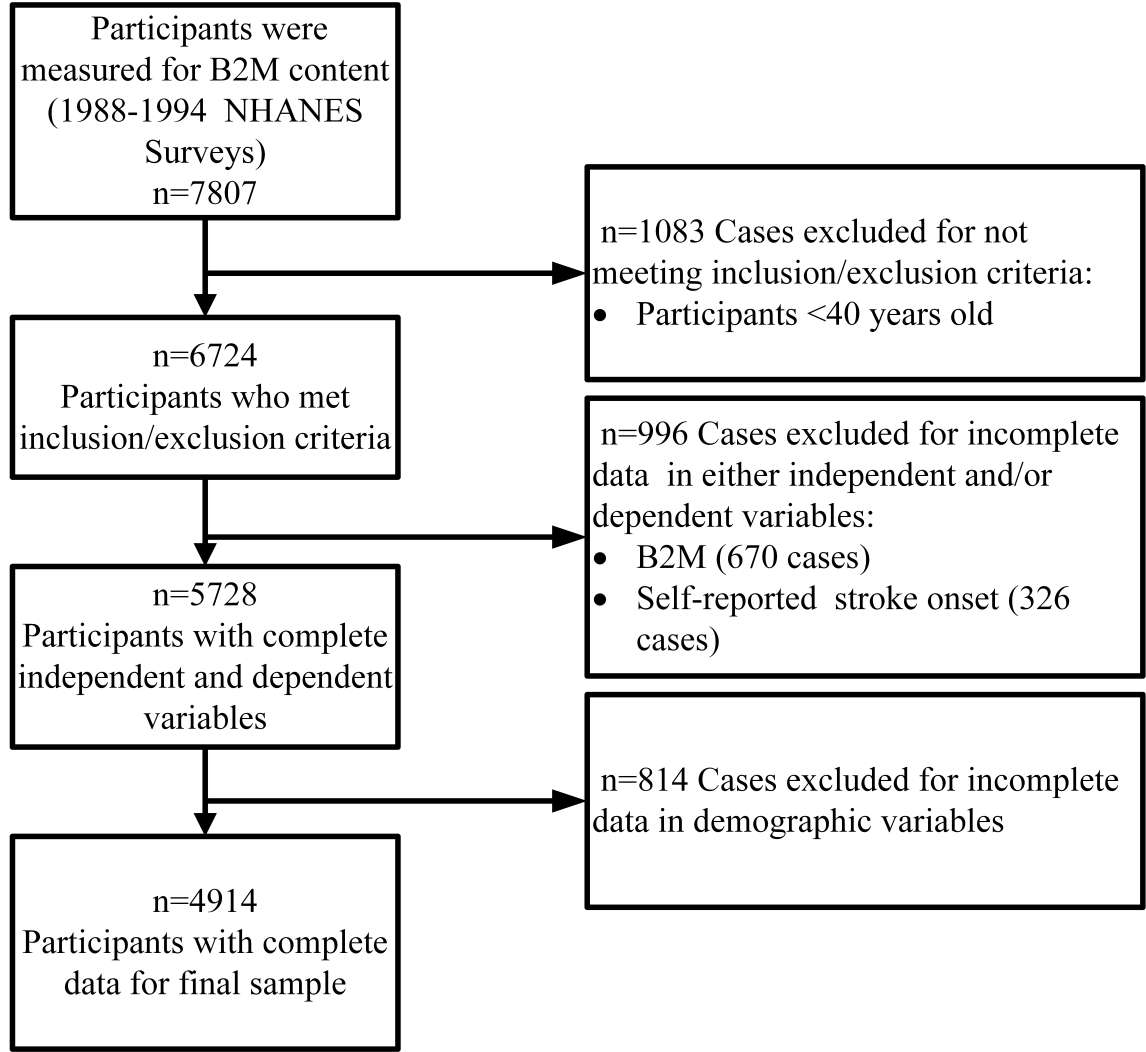
**Flowchart of the study**.

Through sample selection, 4914 patients were finally included in the study (Fig. 1). Table 1 shows baseline characteristics of participants (mean age = 63.0 years, 44.3% male), by quintiles of serum β2M levels. At baseline, participants with higher β2M were more likely to be females, older individuals (≥60 years), drinkers, smokers, or individuals with hypertension and diabetes. They were less likely to be Mexican American, non-married, obese, or with low glycated hemoglobin (%), low serum creatinine, and low c-reactive protein level. Moreover, they were more likely to have lower LDL-cholesterol, HDL-cholesterol, and GFR. The Spearman correlations of β2M with age, BMI, and a range of biochemical factors are shown in Table 2.

**Table 1.** **Baseline demographic characteristics of the study population, according to quartiles of β2M**.

Characteristics		β2M					*p* value
		Q1	Q2	Q3	Q4	Q5	
		<1.73	1.73–2.00	2.01–2.33	2.34–2.90	≥2.91	
Total, N		956	976	996	999	987	
Age							
	<60	64.6	40.2	24.5	12.6	9.1	<0.001
	≥60	35.4	59.8	75.5	87.4	90.9	
Sex							
	Male	45.7	46.1	43.5	45.2	39.8	<0.001
	Female	54.3	53.9	56.5	54.8	60.2	
Race/ethnicity							
	Non-Hispanic white	74.9	83.1	84.3	85.2	85.2	<0.001
	Non-Hispanic black	12.4	8.6	7.8	6.9	8.7	
	Mexican American	4.3	2.8	2.8	2.2	1.9	
	Other	8.4	5.5	5.1	5.6	4.2	
Marital status							
	Married	76.3	73.6	65.8	65.2	55.4	<0.001
	Widowed	7.2	14.3	18.2	23.6	33.8	
	Divorced	10.9	6.5	8.8	6.2	5.3	
	Single	5.6	5.6	7.2	5.0	5.5	
Ratio of family income to poverty							
	<1.58	23.0	23.3	32.8	31.2	43.6	<0.001
	1.58–4.09	45.2	43.7	44.8	45.2	40.1	
	>4.09	31.8	33.0	22.3	23.6	16.2	
BMI, kg/$m^{2}$							
	Normal (<25.0)	44.3	37.0	33.3	28.5	37.0	<0.001
	Over weight (25.0–29.9)	36.2	41.8	39.2	40.7	31.9	
	Obese (≥30.0)	19.5	21.2	27.5	30.8	31.1	
Alcohol							
	Never drinker	44.0	51.9	58.4	64.1	68.1	<0.001
	Moderate drinker	31.0	28.3	20.4	18.3	13.6	
	Heavy drinker	24.9	19.4	19.8	15.8	15.2	
	Missing	0.1	0.4	1.4	1.8	3.1	
Smoking							
	Never smoker	43.6	42.1	44.5	43.5	44.6	<0.001
	Former smoker	32.6	35.8	36.6	43.4	38.8	
	Current smoker	23.8	22.1	18.9	13.1	16.6	
Glycated hemoglobin (%)		5.53 ± 0.04	5.64 ± 0.03	5.62 ± 0.03	5.77 ± 0.03	5.89 ± 0.04	0.030
Serum Creatinine (mg/dL)		1.01 ± 0.01	1.06 ± 0.01	1.11 ± 0.01	1.16 ± 0.01	1.42 ± 0.02	<0.001
LDL-cholesterol (mg/dL)		159.02 ± 3.46	150.36 ± 1.66	158.95 ± 3.01	154.94 ± 2.62	153.38 ± 2.50	0.444
HDL-cholesterol (mg/dL)		54.31 ± 0.54	52.13 ± 0.51	51.89 ± 0.52	48.03 ± 0.46	48.36 ± 0.49	<0.001
C-reactive protein (mg/dL)		0.35 ± 0.01	0.37 ± 0.02	0.46 ± 0.02	0.50 ± 0.02	0.90 ± 0.04	<0.001
GFR		117.53 ± 0.96	106.51 ± 0.83	99.02 ± 0.86	90.20 ± 0.72	75.29 ± 0.97	<0.001
History of hypertension		25.8	30.9	34.2	46.2	58.4	<0.001
History of diabetes		5.4	7.4	7.5	9.7	15.7	<0.001

Abbreviation: BMI, body mass index (calculated as weight in kilograms divided by height in meters squared); β2M, β2-microglobulin; GFR, glomerular filtration rate. Values are weighted mean ± SE for continuous variables or weighted % for categorical variables.

**Table 2.** **Spearman correlations between β2M and other factors**.

Factors	β2M	*p* value
Age	0.537	<0.001
BMI	–0.004	0.767
C-reactive protein	0.187	<0.001
LDL-cholesterol	–0.022	0.115
HDL-cholesterol	–0.126	<0.001
Glycated hemoglobin	0.098	<0.001
Serum Creatinine	0.436	<0.001
eGFR	–0.515	<0.001

### 3.2 Stroke Mortality

**Fig. 2.**
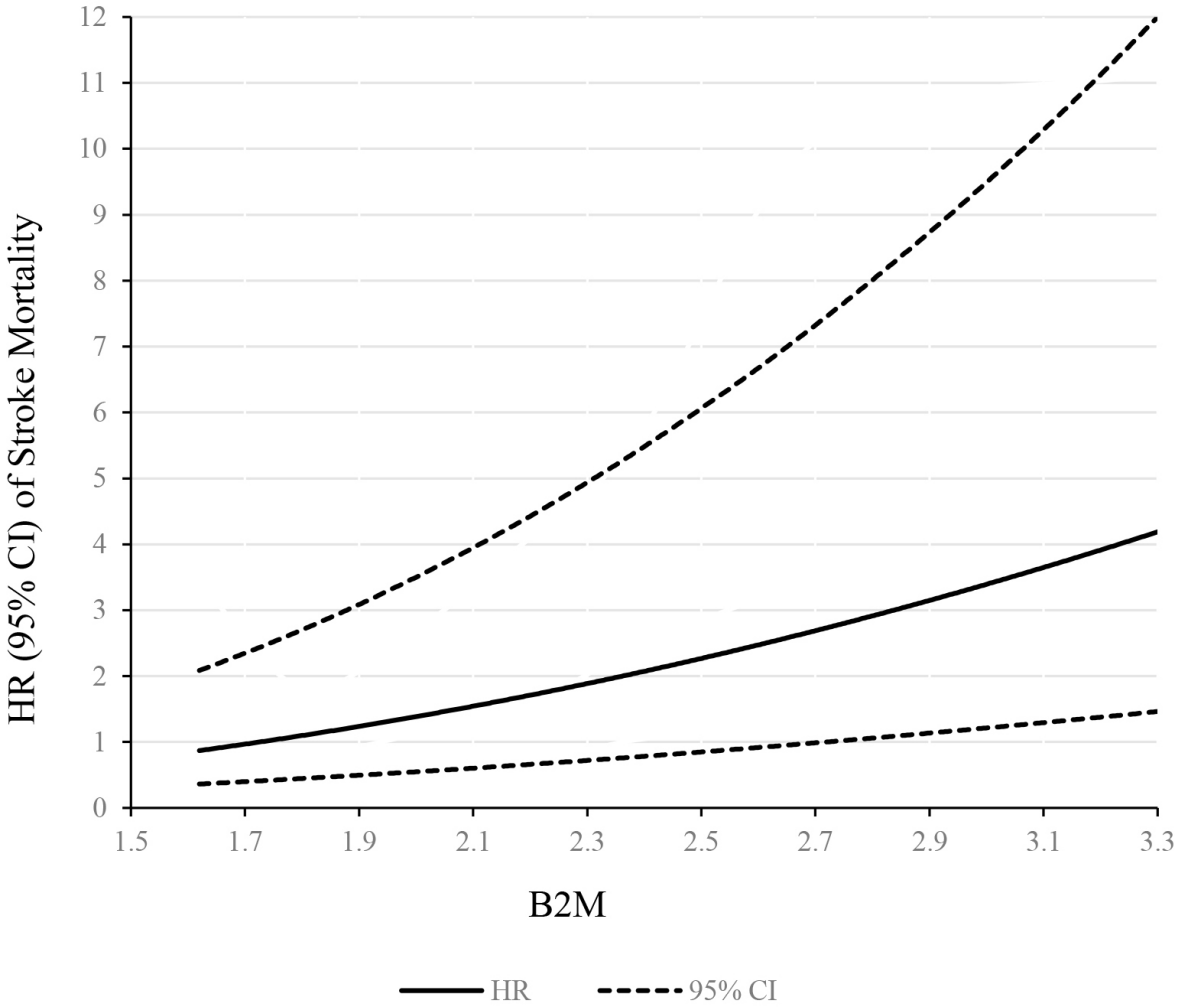
**Dose–response relationship between β2M concentration and stroke mortality**. The solid line and dashed line represent the HRs and their 95% confidence intervals.

Table 3 showed that participants with high β2M levels were at higher risk of stroke mortality in unadjusted model (Q5 VS Q1; HR 6.83, 95% CI 3.10–15.04, *p* for trend < 0.001). After multivariable adjustment, β2M was still statistically significant associated with stroke mortality in differently adjusted model (Q5 VS Q1: HR 3.57 in the model 1; HR 3.50 in the model 2; HR 3.45 in the model 3). Another, a linear dose–response association was observed between β2M levels and risk of stroke mortality (Fig. 2). Stroke mortality risk increased monotonically with β2M modeled continuously, with no indication of a threshold.

**Table 3.** **The association of β2M with stroke and all-cause mortality**.

		β2M					*p* for trend
		Q1	Q2	Q3	Q4	Q5	
		<1.73	1.73–2.00	2.01–2.33	2.34–2.90	≥2.91	
Stroke mortality							
	Deaths, No. (%)	28 (2.4)	46 (3.0)	43 (3.0)	63 (7.0)	74 (6.1)	
	Deaths/person-years	396/19076	648/17460	489/15964	603/12821	577/8633	
	Unadjusted	1 [Reference]	1.43 (0.69, 2.98)	1.65 (0.80, 3.40)	4.98 (2.34, 10.60)	6.83 (3.10, 15.04)	<0.001
	Model 1	1 [Reference]	1.07 (0.52, 2.21)	1.05 (0.51, 2.18)	2.92 (1.28, 6.70)	3.57 (1.70, 7.49)	<0.001
	Model 2	1 [Reference]	1.08 (0.53, 2.21)	1.05 (0.49, 2.24)	2.95 (1.27, 6.88)	3.51 (1.58, 7.78)	<0.001
	Model 3	1 [Reference]	1.06 (0.50, 2.26)	1.08 (0.48, 2.44)	3.03 (1.18, 7.75)	3.46 (1.34, 8.95)	0.001
All-cause mortality							
	Deaths, No. (%)	385 (32.5)	560 (50.6)	698 (65.1)	842 (82.5)	930 (91.6)	
	Deaths/person-years	5622/19076	7688/17460	9013/15964	9193/12821	7357/8633	
	Unadjusted	1 [Reference]	1.80 (1.49, 2.18)	2.70 (2.32, 3.14)	4.42 (2.57, 5.47)	8.05 (6.42, 10.10)	<0.001
	Model 1	1 [Reference]	1.33 (1.11, 1.60)	1.66 (1.42, 1.95)	2.52 (2.11, 3.01)	4.37 (3.47, 5.49)	<0.001
	Model 2	1 [Reference]	1.36 (1.14, 1.63)	1.65 (1.43, 1.91)	2.56 (2.15, 3.04)	4.18 (3.33, 5.26)	<0.001
	Model 3	1 [Reference]	1.36 (1.14, 1.62)	1.69 (1.45, 1.97)	2.60 (2.17, 3.11)	3.96 (3.04, 5.17)	<0.001

1. Percentages and mortality rates were estimated using U.S. population weights. 2. Values are n or weighted hazard ratio (95% confidence interval). Model 1: adjusted for age, sex, race/ethnicity, and marital status. Model 2: model 1 + Ratio of family income to poverty, BMI, alcohol, smoking. Model 3: model 2 + Glycated hemoglobin (%), Serum Creatinine (mg/dL), LDL-cholesterol (mg/dL), HDL-cholesterol (mg/dL), C-reactive protein (mg/dL), GFR, history of hypertension and history of diabetes.

**Fig. 3.**
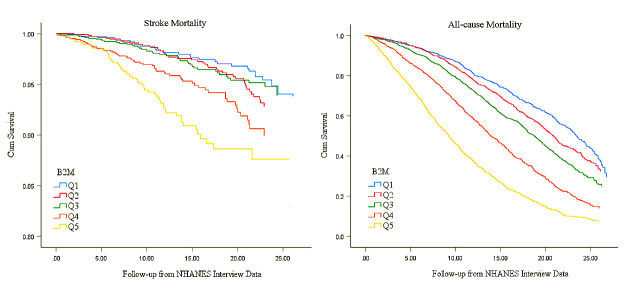
**Adjusted survival curves for stroke and all-cause mortality by quartile**.

As shown in Fig. 3, participants with the highest β2M relative to their lower β2M had much steeper declines in survival over more than 20 years of follow-up. Among participants with the highest β2M at baseline, about 10% for stroke patients had died after 25 years of follow-up. As for the lowest β2M, about only 5% for stroke patients had died.

### 3.3 All-Cause Mortality

**Fig. 4.**
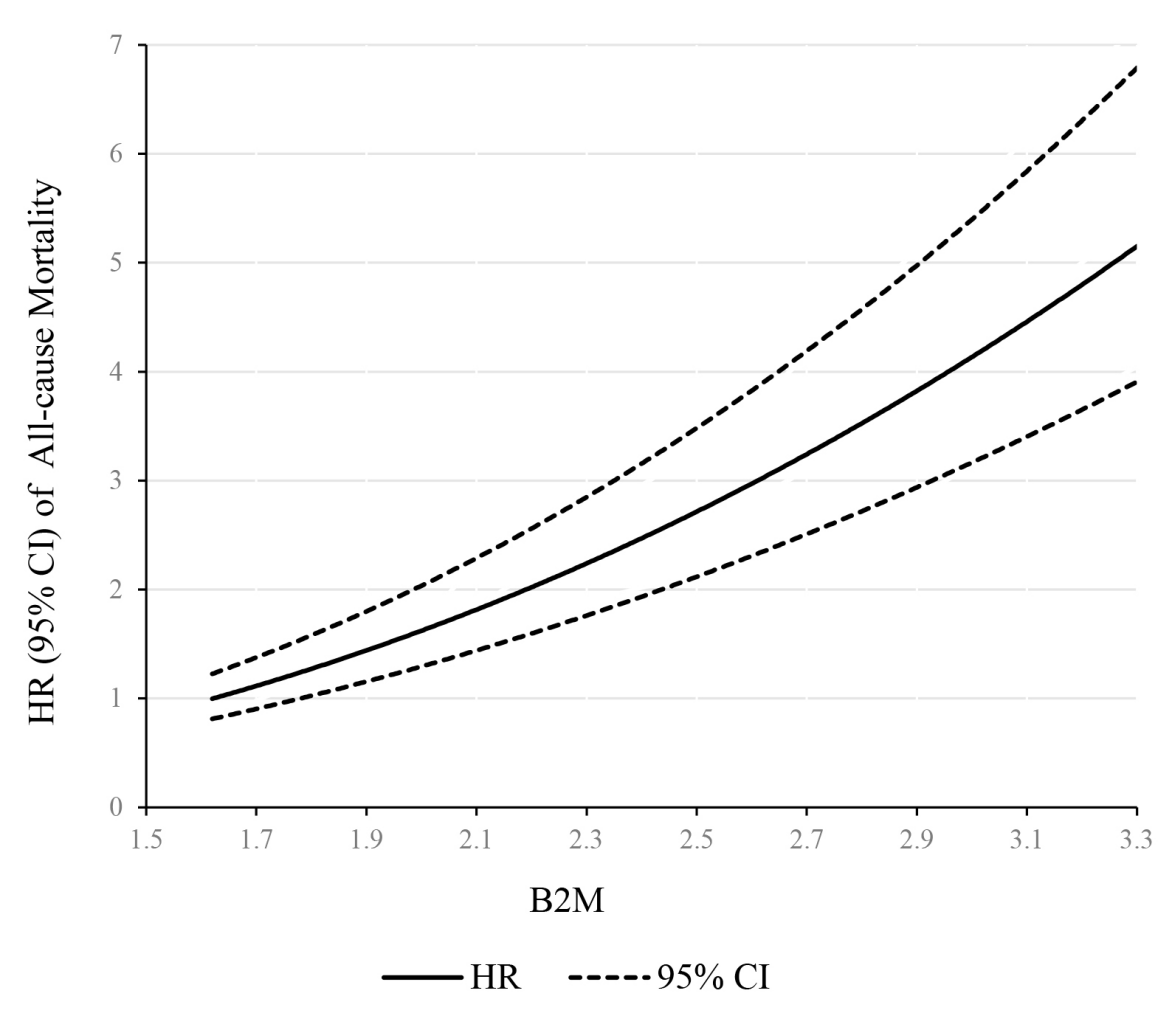
**Dose–response relationship between β2M concentration and all-cause mortality**. The solid line and dashed line represent the HRs and their 95% confidence intervals.

For all-cause mortality, the similar association was observed with β2M. In unadjusted model, β2M was strongly associated with all-cause mortality (Q5 VS Q1; HR 8.05, 95% CI 6.42–10.10, *p* for trend < 0.001). β2M was positively associated with higher all-cause mortality in the age-, sex-, race/ethnicity-, and marital status-adjusted model 1 (Q5 VS Q1; HR 4.37, 95% CI 3.47–5.49; *p* for trend < 0.001). The positive association remained significant after further adjusting for other sociodemographic characteristics and chronic disease (Q5 VS Q1; HR 4.17, 95% CI 3.33–5.21 in the model 2; HR 3.95, 95% CI 3.05–5.12 in the model 3). Also, a linear dose–response association was observed between β2M levels and risk of all-cause mortality (Fig. 4). Mortality risk increased monotonically with β2M modeled continuously, with no indication of a threshold.

As shown in Fig. 3, similar to stroke mortality, participants with the highest β2M relative to their lower β2M had much steeper declines in survival over more than 20 years of follow-up. Among participants with the highest β2M at baseline, about 90% for all-cause mortality had died after 25 years of follow-up. As for the lowest β2M, about 70% for all-cause mortality had died.

### 3.4 Subgroup Analyses

**Fig. 5.**
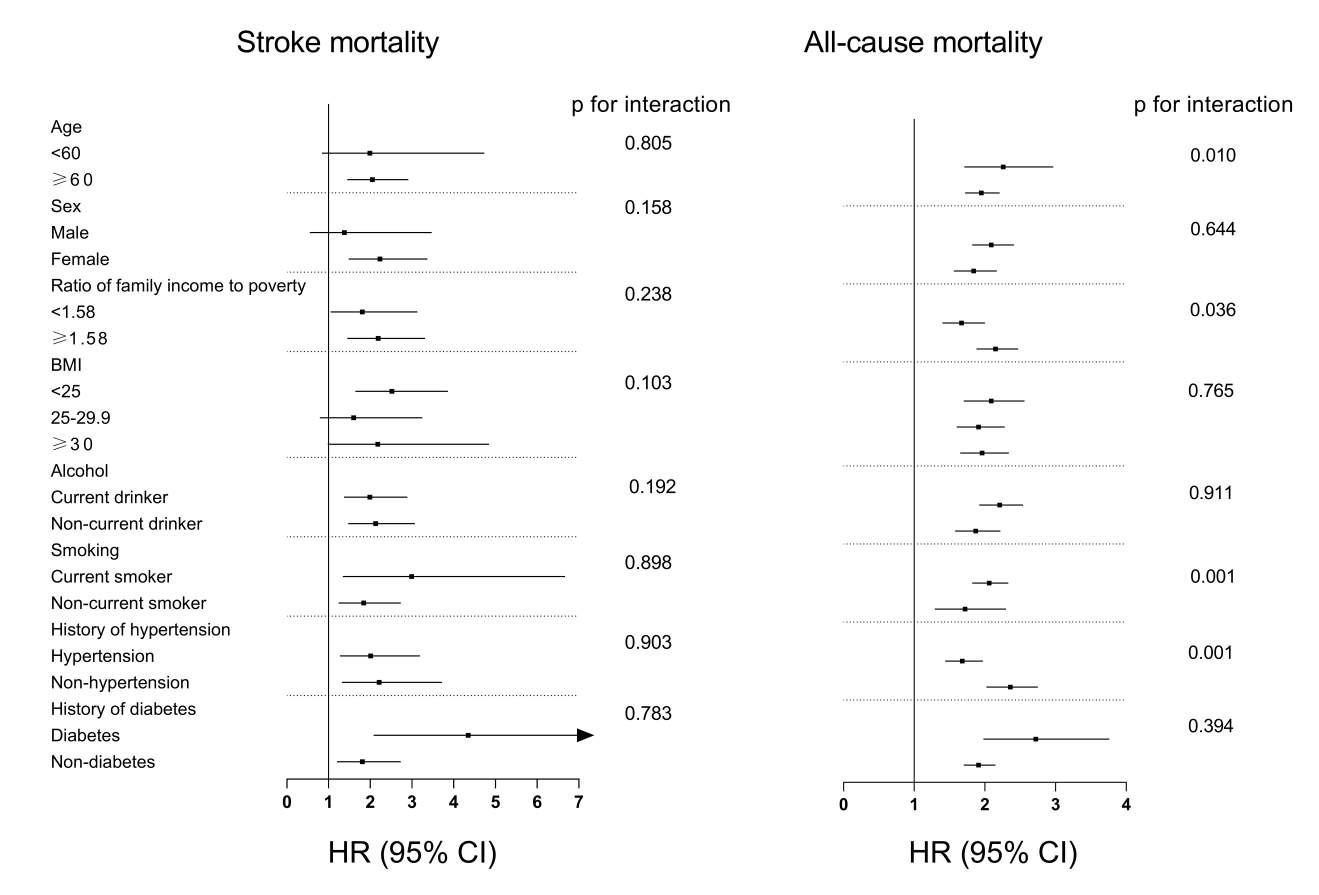
**Subgroup analysis of the association of β2M with stroke and all-cause mortality**.

In the subgroup analyses (Fig. 5), the positive association between concentrations of β2M and stroke mortality was consistent among all participants and all subgroups, and there was no significant difference by varying strata of risk factors for stroke mortality (*p *_interaction_ > 0.05). For all-cause mortality, although the association between β2M and all-cause mortality persisted in all the subgroups, the positive associations were stronger among age <60 years (*p *_interaction_ = 0.010), ratio of family income to poverty ≥1.58 (*p *_interaction_ = 0.036), current smokers (*p *_interaction_ = 0.001), non-hypertensive participants (*p *_interaction_ = 0.001).

### 3.5 Sensitivity Analyses

First, we observed similar associations when we excluded deaths during the first two years of follow-up (**Supplementary Table 1**). Second, there was no significant difference in the association β2M with stroke and all-cause mortality when we excluded participants with histories of chronic kidney disease, diabetes or hypertension disease (**Supplementary Table 2**). Furthermore, we also found unchanged associations when we excluded those with GFR <60 mL/min/1.73 $m^{2}$, the cutoff for abnormal renal function/chronic kidney disease (**Supplementary Table 3**).

## 4. Discussion

This study comprehensively showed that serum β2M concentrations were associated with stroke and all-cause mortality in 4917 U.S. adults aged 40 years or more during a median follow-up of 19.4 years. Also, the association between serum β2M concentrations and stroke mortality was independent of sociodemographic and general stroke risk factors. The risk estimates for the positive association between serum β2M concentrations and all-cause mortality varied the participants’ age, ratio of family income to poverty, smoking status, and history of hypertension.

According to the previous literature, some studies had demonstrated the association between β2M with CVD mortality [10] and stroke incidence [20, 21, 22]; nevertheless, the association between serum β2M and specific stroke mortality had not been investigated. β2M may be a marker of subclinical renal dysfunction and small vessel disease, and is associated with incident peripheral artery disease and severity of peripheral artery disease [9, 10, 11, 12, 13, 14]. β2M levels was elevated with systemic inflammation, due to systemic inflammation, and exacerbated by in-hospital infections, can increase morbidity and mortality in stroke patients [30, 31], suggesting that inflammation may explain some, but not all, of the association between β2M and stroke risk. Furthermore, previous studies indicated that higher β2M levels were associated with 1.3–2 times increased risk of stroke incidence compared with lower β2M levels [22, 32]. In the Risk for Cardiovascular Events Study of 1005 male and female, median follow-up of 3 years, β2M levels (Q4: ≥2.59 VS Q1: ≤1.49 mg/L) was positively associated with an increased risk of stroke, adjusted HR (95% CI) was 1.62 (1.16–2.67) [32]. For the risk of ischemic stroke among 946 women in the Nurses’ Health Study [22], women in the highest quartile (≥2.59 mg/L) of β2M had a statistically significant increase in the odds of developing an ischemic stroke (OR 1.71, 95% CI 1.14–2.56) compared to those in the lowest quartile (≤1.49 mg/L) after adjustment for traditional stroke risk factors. In the Atherosclerosis Risk in Community Study of 8622 men and women, with a median follow-up of 11.9 years [20], β2M levels were associated with significantly increased risk of total stroke in both those with and without chronic kidney disease. The Multivariable HR (95% CI) was 1.16 (1.04–1.30) in those with chronic kidney disease and was 1.30 (1.13–1.49) in those without. This suggests that even among those with “normal” kidney function as indicated by creatinine-based GFR, β2M levels may predict the risk of stroke. However, in this study, we explored the association between β2M and stroke mortality, and expand on previously prior findings from stroke incidence to stroke mortality by demonstrating that baseline β2M levels were associated with risk of stroke mortality, during a median follow-up of 19.4 years. The HR (95% CI) associated with Q5 compared to Q1 of serum β2M was 3.46 (1.34, 8.95).

This study indicated that higher β2M concentrations were positively associated with all-cause mortality. Previous studies have also indicated that the β2M was positively associated with all-cause mortality in participants with chronic kidney disease , HR and 95% CI: 2.52 (1.89, 3.36) [9], chronic obstructive lung disease, HR and 95% CI: 1.09 (1.05, 1.14) [10], diabetes, HR and 95% CI: 7.35 (1.01, 53.38) [33], cancer, HR and 95% CI: 1.25 (1.06–1.47) [34], hemodialysis mortality, HR and 95% CI: 1.09 (1.05–1.14) [35], and CVD, RR and 95% CI: 2.29 (1.51–3.49) [36]. Circulating β2M is a potential biomarker that reflects the oxidative stress, or dialysate contamination, that involved in mucosal immunity, tumor monitoring, immunoglobulin and albumin homeostasis [37]. In addition, the increase of β2M was also closely related to the inflammatory response and the decrease of glomerular filtration rate (GFR) [9, 10, 38, 39]. In addition, other studies also show that a wide range of inflammatory risk factors increase the risk of all-cause mortality [40, 41]. The present study found significant associations between higher β2M levels and increased risks of all-cause mortality even after adjustment for inflammatory markers (e.g., serum creatinine, c-reactive protein, and GFR), which is generally consistent with the results of previous studies [8, 42].

In subgroup analyses, the association of β2M and stroke mortality was independent of sociodemographic and general stroke risk factors (*p *_interaction_ > 0.05). However, the association between β2M with all-cause mortality varied age, ratio of family income to poverty, smoking status, and history of hypertensive, and were stronger in participants with aged <60 years, non-married, ratio of family income to poverty ≥1.58, current smokers, or non-hypertension. A large number of studies have shown that age is closely related to a variety of chronic diseases [43, 44, 45]. However, we found that stronger association between β2M and all-cause mortality in participants aged <60 years in this study. This relationship is not surprising because the study speculated that age may hide the association between β2M and all-cause mortality in participants aged ≥60 years, due to the additional effect of age on all-cause mortality.

The importance of socio-economic status as predictor for all-cause mortality has been emphasized by many studies [24, 46]. High ratio of family income to poverty symbolized low family income. Previous research showed that health inequalities from income inequality exist in low- and middle-income countries as well as in high-income countries [43]. Income fluctuations may have a general impact on health, and may be mediated by physiological changes, psychological changes or health care, including worse mental status, overall quality of life, access to health care, and mortality. Previous studies found that clinical or drug treatment of chronic diseases effectively reduced the concentration of β2M [40, 47, 48]. However, some low-income populations with chronic diseases may abandon clinical or drug treatment and health care services to deal with unexpected financial instability, resulting in high β2M and increased disease risk or disease deterioration.

Smoking and hypertension are strong risk factors of mortality in the United States [49, 50]. Our study found stronger significant association with β2M and all-cause mortality in current smokers, which was consistent with Paweł Wańkowicz’s current study [3]. However, the detailed impact mechanism is still unclear and needs further exploration. To the contrary, we observed a stronger association of β2M with all-cause mortality in non-hypertensive participants than hypertensive participants. There are two possible reasonable explanations for this result. First of all, patients with hypertension may suffer from other chronic diseases before treatment, such as hyperlipidemia, atherosclerosis, and diabetes. Therefore, effective prevention and treatment of risk factors such as blood glucose, blood lipid and blood pressure for patients with chronic diseases and general healthy people can reduce the disease burden and mortality in the future [50]. Second, previous studies indicated that blood pressure treatment was closely associated with serum β2M concentrations, effectively reducing the concentration of β2M [47, 51, 52]. Therefore, we speculate that the changes of function and physiological tissue structure in hypertensive population may mask the association of β2M and all-cause mortality due to additional blood pressure treatment.

In summary, the evidences provided in this study showed the importance of β2M to overall health, especially cerebrovascular disease health, and supported that β2M is an effective predictor of stroke and all-cause mortality. In recent years, as a rare cause, blood elements are closely related to the occurrence of stroke, but the association between β2M and stroke mortality is relatively lacking, so our research will help to further explain the relationship between blood diseases and stroke to some extent. Additionally, β2M may participate in the inflammatory response in the humoral microenvironment and interact with other antigenic bodies or immune molecules. Therefore, it is necessary to further explore the mechanism of β2M and its therapeutic effect in clinical practice. Considering that the pathophysiology, prognosis and clinical characteristics of acute small vessel ischemic stroke are different from other types of cerebral infarction, and lacunar infarction is the stroke subtype with the best functional prognosis. An indispensable research in the future will be to evaluate the correlation between β2M and lacunar and non lacunar acute stroke.

## 5. Conclusions

High β2M levels were associated with an increased risk of stroke and all-cause mortality in U.S. adults of aged ≥40 year in this study. Future studies are needed to further elucidate the mechanisms by which β2M increases the risk of stroke and all-cause mortality, to measure changes in β2M levels during follow-up, and to determine whether β2M levels can be modified through interventions.

## 6. Strengths and Limitations

The strengths of our study included the use of a multi-stage, complex prospective cohort study based on population aged ≥40 years in the U.S., the combined large sample size, and the long follow-up period. In addition, when the study excluded deaths during the first 2 years of follow-up, participants with major chronic diseases (hypertension and diabetes mellitus) at baseline, and GFR <60 mL/min/1.73 $m^{2}$, the associations have not changed substantially, indicating the obvious robustness of our results, and the statistical type II error minimized.

The study also needs to acknowledge several limitations. First, the serum β2M concentration was measured only at baseline, and participants’ serum β2M may have changed during follow-up due to changes in living habits and diet. Second, residual confounding was likely, although a number of covariates were adjusted (for example, aspirin use, hormone replacement therapy, and dietary pattern). Third, mortality outcomes were determined through linkage to the National Death Index with a probabilistic matching algorithm to determine the mortality status that may result in some misclassification, which may exaggerate or narrow the observed findings. Fourth, due to the limitation of death data, the study did not analyze stroke subtypes, such as ischemic and hemorrhagic strokes.

## Data Availability

The data of this study are based on NHANES. Data used were derived from de-identified and publicly database, and More information can be found at (https://wwwn.cdc.gov/nchs/nhanes/nhanes3/DataFiles.aspx).

## Abbreviations

NHANES, the National Health and Nutrition Examination Survey; NCHS, the National Center for Health Statistics; CVD, cardiovascular disease; β2M, serum beta-2 microglobulin; BMI, body mass index; FFQ, food frequency questionnaire; HEI-2010, the Healthy Eating Index-2010; ICD-10, the International Classification of Diseases, 10th revision; HR, hazard ratio; CI, 95% confidence interval; Q1, lower quartile; Q2, second quartile; Q3, third quartile; Q4, fouth quartile.

## Supplementary Material

Supplementary material associated with this article can be found, in the online version, at https://doi.org/10.31083/j.rcm2402043.
