# Supplementary material for: Association of Beta-2 Microglobulin with Stroke and All-Cause Mortality in Adults Aged ≥40 in U.S.: NHANES III
Source: Rev Cardiovasc Med. 2023 Feb 2;24(2):43. doi: 10.31083/j.rcm2402043 (PMC11273124; doi:10.31083/j.rcm2402043)
Supplement: Supplementary file 1 [file 2153-8174-24-2-043-s1.zip › Supplementary table 1.docx]

|  | |  | | B2M | | | | |
| --- | --- | --- | --- | --- | --- | --- | --- | --- |
|  |  | Q1  <1.73 | Q2  1.73-2.00 | | Q3  2.01-2.33 | Q4  2.34-2.90 | Q5  ≥2.91 | *P trend* |
| Stroke mortality |  | | | | | | | |
| Deaths, No. (%) | | 27(2.4) | 46(3.0) | | 42(3.0) | 56(6.2) | 66(5.7) |  |
| Unadjusted | | 1.00 [Reference] | 1.45(0.69,3.02) | | 1.67(0.80,3.46) | 4.45(2.06,9.62) | 6.80(3.00,15.42) | <0.001 |
| Model1 | | 1.00 [Reference] | 1.08(0.52,2.24) | | 1.06(0.51,2.21) | 2.62(1.11,6.19) | 3.50(1.63,7.51) | <0.001 |
| Model2 | | 1.00 [Reference] | 1.10(0.54,2.25) | | 1.05(0.50,2.22) | 2.62(1.11,6.17) | 3.39(1.50,7.63) | <0.001 |
| Model3 | | 1.00 [Reference] | 1.08(0.51,2.29) | | 1.08(0.48,2.42) | 2.70(1.03,7.02) | 3.38(1.29,8.88) | <0.001 |
| All-cause mortality |  | | | | | | | |
| Deaths, No. (%) ^a^ | | 385(32.5) | 560(50.6) | | 698(65.1) | 842(82.5) | 930(91.6) |  |
| Unadjusted | | 1.00 [Reference] | 1.83(1.49,2.25) | | 2.72(2.33,3.18) | 4.42(3.48,5.61) | 7.72(6.10,9.75) | <0.001 |
| Model1 | | 1.00 [Reference] | 1.35(1.11,1.65) | | 1.68(1.43,1.97) | 2.52(2.07,3.08) | 4.18(3.29,5.30) | <0.001 |
| Model2 | | 1.00 [Reference] | 1.38(1.14,1.68) | | 1.66(1.43,1.92) | 2.55(2.11,3.09) | 3.96(3.12,5.04) | <0.001 |
| Model3 | | 1.00 [Reference] | 1.37(1.13,1.66) | | 1.69(1.46,1.97) | 2.57(2.12,3.11) | 3.75(2.86,3.11) | <0.001 |

**Supplementary Table 1** Sensitivity Analysis for Stroke and All-cause Mortality Associated with B2M, Excluding Deaths During the First 2 Years of Follow-up

1.Percentages and mortality rates were estimated using US population weights.

2.Values are n or weighted hazard ratio (95% confidence interval). Model 1: adjusted for age, sex, race/ethnicity. Model 2: model 1 +marital status, ratio of family income to poverty, BMI, alcohol, and smoking. Model 3: model 2 + Glycated hemoglobin (%), Serum Creatinine (mg/dL), LDL-cholesterol (mg/dL), HDL-cholesterol (mg/dL), C-reactive protein(mg/dL), GFR, history of hypertension and history of diabetes mellitus.
