# Supplementary material for: Association of Beta-2 Microglobulin with Stroke and All-Cause Mortality in Adults Aged ≥40 in U.S.: NHANES III
Source: Rev Cardiovasc Med. 2023 Feb 2;24(2):43. doi: 10.31083/j.rcm2402043 (PMC11273124; doi:10.31083/j.rcm2402043)
Supplement: Supplementary file 1 [file 2153-8174-24-2-043-s1.zip › Supplementary table 2.docx]

**Supplementary table 2** Sensitivity Analysis for Stroke and All-cause Mortality Associated with B2M, Excluding Participants with Histories of Disease (CKD, Hypertension or Diabetes)

|  | |  | | B2M | | | | |
| --- | --- | --- | --- | --- | --- | --- | --- | --- |
|  |  | Q1  <1.73 | Q2  1.73-2.00 | | Q3  2.01-2.33 | Q4  2.34-2.90 | Q5  ≥2.91 | *P trend* |
| Stroke mortality |  | | | | | | | |
| Deaths, No. (%) | | 16(1.9) | 26(3.3) | | 19(1.1) | 31(7.9) | 21(4.4) |  |
| Unadjusted | | 1.00 [Reference] | 2.07(1.01,4.22) | | 0.76(0.33,1.72) | 7.20(2.81,18.42) | 6.58(2.35,18.44) | <0.001 |
| Model1 | | 1.00 [Reference] | 1.45(0.67,3.13) | | 0.43(0.16,1.12) | 3.74(1.15,12.13) | 3.57(1.23,10.40) | 0.003 |
| Model2 | | 1.00 [Reference] | 1.50(0.74,3.04) | | 0.42(0.16,1.11) | 3.85(1.31,11.32) | 3.30(1.05,8.57) | 0.006 |
| Model3 | | 1.00 [Reference] | 1.41(0.69,2.89) | | 0.38(0.14,0.99) | 3.47(1.30,9.25) | 3.59(1.02,10.27) | 0.005 |
| All-cause mortality |  | | | | | | | |
| Deaths, No. (%) ^a^ | | 213(27.7) | 307(45.1) | | 386(60.1) | 399(78.0) | 355(89.2) |  |
| Unadjusted | | 1.00 [Reference] | 1.89(1.46,2.45) | | 2.92(2.38,3.58) | 4.73(3.55,6.30) | 8.85(6.49,12.07) | <0.001 |
| Model1 | | 1.00 [Reference] | 1.35(1.04,1.76) | | 1.69(1.34,2.14) | 2.54(1.92,3.36) | 4.88(3.62,6.59) | <0.001 |
| Model2 | | 1.00 [Reference] | 1.40(1.09,1.80) | | 1.65(1.33,2.05) | 2.61(2.03,3.34) | 4.31(3.21,5.78) | <0.001 |
| Model3 | | 1.00 [Reference] | 1.43(1.11,1.85) | | 1.74(1.39,2.19) | 2.84(2.18,3.70) | 5.29(3.77,7.41) | <0.001 |

CKD chronic kidney disease

1.Percentages and mortality rates were estimated using US population weights.

2.Values are n or weighted hazard ratio (95% confidence interval). Model 1: adjusted for age. Model 2: model 1 +sex, race/ethnicity, marital status, ratio of family income to poverty. Model 3: model 2 + BMI, alcohol, smoking, Glycated hemoglobin (%), Serum Creatinine (mg/dL), LDL-cholesterol (mg/dL), HDL-cholesterol (mg/dL), C-reactive protein(mg/dL), and GFR.
