# Supplementary material for: Association of Beta-2 Microglobulin with Stroke and All-Cause Mortality in Adults Aged ≥40 in U.S.: NHANES III
Source: Rev Cardiovasc Med. 2023 Feb 2;24(2):43. doi: 10.31083/j.rcm2402043 (PMC11273124; doi:10.31083/j.rcm2402043)
Supplement: Supplementary file 1 [file 2153-8174-24-2-043-s1.zip › Supplementary table 3.docx]

|  | |  | | B2M | | | | |
| --- | --- | --- | --- | --- | --- | --- | --- | --- |
|  |  | Q1  <1.73 | Q2  1.73-2.00 | | Q3  2.01-2.33 | Q4  2.34-2.90 | Q5  ≥2.91 | *P trend* |
| Stroke mortality |  | | | | | | | |
| Deaths, No. (%) | | 28(2.4) | 46(3.0) | | 40(2.8) | 59(7.3) | 41(5.9) |  |
| Unadjusted | | 1.00 [Reference] | 1.45(0.70,3.03) | | 1.59(0.78,3.22) | 5.16(2.43,10.98) | 6.16(2.76,13.72) | <0.001 |
| Model1 | | 1.00 [Reference] | 1.08(0.53,2.23) | | 1.00(0.49,2.00) | 3.00(1.31,6.84) | 3.11(1.55,6.23) | <0.001 |
| Model2 | | 1.00 [Reference] | 1.09(0.54,2.22) | | 0.99(0.47,2.09) | 3.03(1.32,6.97) | 3.05(1.42,7.71) | <0.001 |
| Model3 | | 1.00 [Reference] | 1.08(0.52,2.26) | | 1.07(0.47,2.41) | 3.51(1.42,8.68) | 3.97(1.55,10.21) | <0.001 |
| All-cause mortality |  | | | | | | | |
| Deaths, No. (%) ^a^ | | 384(32.5) | 559(50.9) | | 677(65.8) | 786(82.1) | 597(90.3) |  |
| Unadjusted | | 1.00 [Reference] | 1.83(1.51,2.21) | | 2.76(2.37,3.21) | 4.37(3.52,5.43) | 7.23(5.57,9.38) | <0.001 |
| Model1 | | 1.00 [Reference] | 1.34(1.11,1.62) | | 1.67(1.42,1.97) | 2.46(2.04,2.98) | 3.93(2.99,5.16) | <0.001 |
| Model2 | | 1.00 [Reference] | 1.37(1.14,1.65) | | 1.66(1.44,1.91) | 2.52(2.11,3.01) | 3.76(2.88,4.91) | <0.001 |
| Model3 | | 1.00 [Reference] | 1.39(1.15,1.67) | | 1.78(1.52,2.09) | 2.78(2.30,3.36) | 4.11(2.98,5.67) | <0.001 |

**Supplementary table 3** Sensitivity Analysis for Stroke and All-cause Mortality Associated with B2M, Excluding eGFR < 60 ml/min/1.73 m^2^

1.Percentages and mortality rates were estimated using US population weights.

2.Values are n or weighted hazard ratio (95% confidence interval). Model 1: adjusted for age, sex, race/ethnicity. Model 2: model 1 +marital status, ratio of family income to poverty, BMI, alcohol, and smoking. Model 3: model 2 + Glycated hemoglobin (%), Serum Creatinine (mg/dL), LDL-cholesterol (mg/dL), HDL-cholesterol (mg/dL), C-reactive protein(mg/dL), GFR, history of hypertension and history of diabetes mellitus.
